# Online advertisement: how are visual strategies affected by the distance and the animation of banners?

**DOI:** 10.3389/fpsyg.2014.00211

**Published:** 2014-03-17

**Authors:** Léa Pasqualotti, Thierry Baccino

**Affiliations:** Laboratory of Human and Artificial Cognition, Department of Psychology, CHArt/LUTIN, University of Paris VIIISaint-Denis, France

**Keywords:** visual strategies, web pages, advertising, banner blindness

## Abstract

Most of studies about online advertisements have indicated that they have a negative impact on users' cognitive processes, especially when they include colorful or animated banners and when they are close to the text to be read. In the present study we assessed the effects of two advertisements features—distance from the text and the animation—on visual strategies during a word-search task and a reading-for-comprehension task using Web-like pages. We hypothesized that the closer the advertisement was to the target text, the more cognitive processing difficulties it would cause. We also hypothesized that (1) animated banners would be more disruptive than static advertisements and (2) banners would have more effect on word-search performance than reading-for-comprehension performance. We used an automatic classifier to assess variations in use of *Scanning* and *Reading* visual strategies during task performance. The results showed that the effect of dynamic and static advertisements on visual strategies varies according to the task. Fixation duration indicated that the closest advertisements slowed down information processing but there was no difference between the intermediate (40 pixel) and far (80 pixel) distance conditions. Our findings suggest that advertisements have a negative impact on users' performance mostly when a lots of cognitive resources are required as for reading-for-comprehension.

## Introduction

Because the economic model of the Internet is based on advertisements, advertisers attempt to grab users' attention by any means possible. Even during an activity such as reading, advertisements can disrupt the attention of readers, making text comprehension more difficult (Baccino, 2004). However, attention is a highly labile capacity and reports of attentional disturbance from online advertisements have led to extensive research on the influence of banners on Internet users (Diaper and Waelend, 2000; Burke et al., 2005; Pagendarm and Schaumburg, 2006; Zhang, 2006; Simola et al., 2011). Studies can be classified according to whether they focused on the level of control of attentional processes (Theeuwes, 1994; Theeuwes and Burger, 1998; Drèze and Hussherr, 2003; Stenfors et al., 2003) or the distinction between overt and covert attention (Burke et al., 2005; Diaper and Waelend, 2000; Simola et al., 2011).

### Shifts of attention

Studies of online advertisement have retained the classical distinction between automatic and controlled attentional processes (Theeuwes, 1994; Theeuwes and Burger, 1998; Drèze and Hussherr, 2003; Stenfors et al., 2003; Simola et al., 2011). From a bottom–up perspective, involuntary shifts of attention are guided by salient elements of the on-screen display (Itti and Koch, 2000). Controlled shifts of attention to particular elements of the interface are determined by the goal: top–down processing. Nevertheless, research has provided evidence for a two-component model of attentional shifting: a fast bottom–up process and a slower top–down mechanism (Braun and Sagi, 1990; Hikosaka et al., 1996; Braun, 1998; Braun and Julesz, 1998; Itti and Koch, 2000). This research has also distinguished between overt attention and covert attention. Overt attentional shifts are manifested as an eye movement toward the element which has grabbed the individual's attention. Covert attentional shifts do not involve eye movement. Previous work has provided mixed results on overt and covert shifts of attention, for example Burke et al. (2005) suggested that online advertisements affects users' performance even when they do not show eye movement or overt attention, however, Simola et al. (2011) obtained data which indicated that users directly fixated advertising banners, particularly those on the right-hand side of Web pages.

### Impact of advertisements on attention

According to Kahneman's theory of attention (1973), sharing capacity is reduced when one of two competing tasks is highly demanding. Based on this theory, Simola et al. (2011) suggested that advertisements act as distractors: covertly attending to advertisements decreases the cognitive resources assigned to the main task. Whether attention to advertisements is overt or covert, controlled or automatic, there is a consensus that online advertisements affect users' performance. Recent results (Simola et al., 2011) suggested that users paid overt attention to banners, particularly when they were located on the right-hand side of a Web page. These authors specified that the most distracting Web page configuration was characterized by a static banner at the top of the page and an animated banner on the right-hand side. Previous work also highlighted the impact of the size of advertisements or advertisement elements on attentional shifts, showing that larger advertisements attract more fixations (Lohse, 1997; Wedel and Pieters, 2007). Other studies have linked larger surface size to higher visual saliency (Pieters et al., 2007; Orquin et al., 2012). Previous research has also consistently found that the impact of online advertisement varies according to the task: tasks which require higher level cognitive resources and deeper information processing suffer less interference from advertisements (Diaper and Waelend, 2000; Pagendarm and Schaumburg, 2006; Simola et al., 2011). Additionally, Wang and Day (2007) reported that the level of attention paid to an online advertisement varies according to the stage of the task; they found that users were more sensitive to banners at the beginning and the end of an information search task.

### Banner blindness

Not all the research has confirmed the hypothesis that online advertisements affect users' performance; some studies found that some users' ignore the banners (Benway and Lane, 1998; Drèze and Hussherr, 2003; Stenfors et al., 2003). This capacity actively to ignore advertisements—which are typically salient elements of a visual display—is called “banner blindness” and was first reported by Benway and Lane (1998). These authors investigated how users browsed through a corporate Intranet to find a link to Internet courses. They reported that even large, colorful or dynamic banners which may contain information relevant to the task can be ignored. However, Benway and Lane (1998) did not used actual advertising banners but banner advertisement style-links. Previous studies have also indicated that the position of advertisements affects the strength of banner blindness (Burke et al., 2005; Cooke, 2008; Owens et al., 2011). Cooke (2008) and Owens et al. (2011) obtained similar results which indicated that users actively ignored the right-hand side of Web pages when they expected to find an advertisement there. It was suggested that users may anticipate the position of the banners and may respond by focusing on the top of the page (Burke et al., 2005). Owens et al. (2011) also suggested that users tend actively to ignore areas of the interface where advertisements are usually located. However, Theeuwes and Burger (1998) reported that the banner blindness phenomenon only occurs when users are aware of the distractor and its features. These authors also stated that the phenomenon disappears when the distractor varies randomly from one trial to another.

### Objectives of the present study

The objective of the present study was to assess the impact of banners on two types of visual strategy used for visual inspection: reading and scanning. We investigated the impact of distance from the target material and animation of advertising banners on visual strategies. We investigated two specific questions. (1) In which conditions do we observe a banner blindness phenomenon? (2) How do visual strategies vary with distance from the target and animation of banners? We recorded participants' eye movements while they performed two reading different activities. Participants performed trials of a word-search task and a reading-for-comprehension task in random order. The goal of the word-search task was to find a specific target word in a Web page. The reading-for-comprehension task required participants to scan or read the Web page attentively in order to summarize the topic afterwards. It was hypothesized that the closer the advertisement was to the target, the more difficulty participants would have with task processing. We also hypothesized that animated banners would be more disrupting than static advertisements. We predicted that participants would be disturbed by advertisements while performing the word-search task; because the reading-for-comprehension task required more cognitive resources, we predicted that participants would apply strategies to ignore the banners and would not be distracted by them. Previous studies have showed that readers can switch between different cognitive states whilst performing a reading activity, for instance shifting between scanning and reading (Carver, 1990; Simola et al., 2008; Cole et al., 2011). These different cognitive states can be identified by specific eye movement patterns (Lemaire et al., 2011).

Our study attempted to classify visual strategies automatically. The classification data were used to explore how the effect of advertising banners on visual strategies varies according to the depth of processing required by the target task and how advertisements generate task-switching. From a theoretical standpoint, the present study potentially provides new perspectives on theories on online advertising and attention and the methodologies used to investigate online attention. From a practical standpoint, information on the effects of advertising banners could guide Web designers, developers and advertisers in their choice of banners distance and animation.

## Experiment

### Participants

The required sample size for *F*-tests (repeated measures ANOVA, within-subjects factors) was estimated by a power analysis (GPower 3.1.7) (Faul et al., 2007). The results showed that with 12 experimental conditions (see below for the Design) and 24 trials, 12 participants would be required to achieve a significance level of *p* = 0.05 (power = 0.95; effect size = 0.25). Twenty-four participants (12 females, 12 males, all right-handed) were tested. The participants were students at the University of Paris VIII and the Ecole Pratique des Hautes Etudes (EPHE). Their mean age was 30 years; the range was from 21 to 38 years. All participants were native French speakers and reported normal or corrected to normal vision. They were not aware of the purpose of the study. The students did not receive any reward for their participation.

### Apparatus

Eye movements were recorded using an infrared video eye-tracking device (SMI RED500, SensoMotoric Instruments, Teltow: Germany) sampling pupil and corneal reflection at 500 Hz. The screen coordinates of the left eye were sampled. The system has a spatial tracking accuracy of approximately 0.5° of visual angle. The calibration was run on 9 points to optimize spatial tracking accuracy. Drift was corrected once during the experiment, after 12 trials. Data were recorded with Experiment Center software (SMI Teltow, Germany) and processed with BeGaze software.

The participants were seated on a chair at a fixed distance of approximately 57 cm away from the monitor and the eye-tracker. A chin-rest was used to minimize head movements during the recording. Participants were given the opportunity to adjust the seat and chin-rest to the most comfortable position. The stimuli were presented on a 24” Dell 2007 FP LCD flat screen with a 60 Hz refresh rate. The screen resolution was 1280^*^1024 pixels. With this screen resolution and the given distance from the screen, 1° of visual angle encompassed 2.3 letters on average.

### Stimuli

#### Texts

Fifty texts from six domains—France, World, Science, Technology, Sport, and Culture—were extracted from newspaper websites. The length of the texts was controlled by the number of words (*M* = 168.63; *SD* = 4.85) and the number of lines (*M* = 12.25; *SD* = 0.59). The 50 texts were pretested to ensure that the texts used in the main task all had a similar level of difficulty. Eight students from the University of Paris VIII participated in the pretest. The relative difficulty of each text was evaluated with 3 subjective questions and 3 inferential questions. For the subjective questions, participants rated the text difficulty using a five-point Likert scale (from “1”—very difficult to “5”—very easy). The inferential questions were true-false questions and a correct response required use of information from the texts and participants' general knowledge. Texts were excluded if an error was made on the inferential questions and if the mean rating was ≤2 on the Likert scale. Four training texts and 24 experimental texts were selected and integrated into Web-like pages that we created. The average estimated difficulty of the 28 texts was about 3.92 (*SD* = 0.59).

#### Web pages

**Figure 1:**
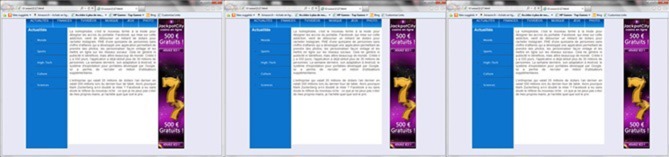
**Examples of Web pages in the three *Distance* conditions (near, 0 px; intermediate, 40 px and far, 80 px)**.

We designed 28 Web-like pages structured as follows (see Figure 1): a horizontal main menu on top of the page, a vertical menu on the left-hand side and a central text. An advertising banner was positioned on the right-hand side of the 24 experimental pages. There were 3 possible distances (in pixels; px) between the text and the banner: 0 px (*near*), 40 px (*intermediate*), and 80 px (*far*). The web pages were stored on a server using FileZilla Client freeware and displayed using the Internet Explorer 9 browser.

#### Banners

Forty-two vertical advertising banners were selected from various websites. In order to control the impact of surface size on attention all banners used the same 120^*^600 px format (Peschel and Orquin, 2013). The visual salience of the banners was also controlled using the Itti and Koch algorithm (Itti and Koch, 2000). Twenty-four banners with similar salience maps were chosen and integrated into the Web pages. The salience maps were compared pairwise in terms of the Area Under Curve (AUC) for each banner (Le Meur and Baccino, 2012); the average correlation was highly significant (*r* = 0.81 *p* < 0.001). Dynamic and static versions of each banner were available.

#### Target words for the word-search task

A single target word per text was selected for the word-search task. Only nouns were chosen. The target was randomly chosen from the beginning, the middle or the end of the text contained in the Web pages. The horizontal position of the target words also varied: they were chosen from the beginning, the middle or the end of the lines. The selected word only appeared once in the text. The target words were 5–8 letters long—this length was selected so that the length of the target words would be close to the mean length of French words. We computed the frequency of the targets using a corpus of French texts (New et al., 2001). The average frequency^1^ was about 81.22 per million (*SD* = 60.68). When displayed on the screen, the target words were 1.2–1.8 cm long, that is, they subtended 1.2–1.8° of visual angle. SMI Experiment Center software allows the user to specify the triggers which advance the task from one trial to another; Areas of Interest (AOIs) can be used as triggers. We defined the target words as AOI triggers to ensure that participants always located the targets and completed the task. We defined a 1000 ms threshold for the time clock of the trigger AOIs, i.e., the participants had to fixate the target words for 1000 ms to access the next trial.

#### Post-test questionnaire

A post-test questionnaire was developed to assess how participants felt affected by the banners. The questionnaire consisted of 14 statements which were evaluated using a five-point Likert scale. Participants completed the questionnaire at the end of the experiment. Half the questions investigated whether participants had paid attention to the advertisements. The other questions evaluated whether participants felt distracted by the advertisements while they were performing the tasks.

### Classifier algorithm

In previous work we developed an algorithm (in PERL) for categorizing fixations in terms of function: *Scanning* or *Reading*. The algorithm accuracy has been evaluated using a classifier technique (Naive Bayes) showing a cross-validation accuracy of 57% for predicting “reading fixations” and 79% for scanning fixations. The classification of a fixation is a function of the orientation *O*, horizontality *H* and the size of the saccade *S* that produced the current fixation (Equation 1).

(1) Class(fixation)=f(O(fixation), H(fixation), S(fixation))

The orientation *O* is obtained by computing the difference between the x-coordinates of the fixation *f* and the previous fixation (Equation 2). A positive result corresponds to a forward saccade and a negative result corresponds to a backward saccade.

(2) $$O\text{(}f\text{) = }x_{f}-\;x_{f}{}_{-\text{1}}$$

The horizontality *H* is the absolute value of the difference between the y-coordinates of the fixation and the previous fixation (Equation 3). We defined a threshold for the horizontality of a saccade: in terms of the height of the white space between two lines of characters: if a saccade was confined within a 45 px vertical gap it was classified as a horizontal saccade.

(3) $$H\text{(}f\text{) = |}y_{f}-\;y_{f-\text{1}}\text{|}$$

The size *S* is the Euclidian distance between the fixation and the previous one (Equation 4). Saccades were classified as short or long by reference to a threshold specified in terms of perceptual span which extends about 12–15 characters to the right side of the fixation point and about 4 characters to the left side (McConkie and Rayner, 1975, 1976), i.e., 4° of visual angle.

(4) $$S(f)=\surd \left({\left(x_{f}-x_{f-1}\right)}^{2}+{\left(y_{f}-y_{f-1}\right)}^{2}\right)$$

Although the most important part of visual information is processed within the foveal region, during reading information is also extracted from the parafoveal region. This corresponds to a perceptual span which is about 4° of visual angle. At a distance of 57 cm from the screen, 1° of visual arc corresponds to 1 cm. With a screen resolution of 1280^*^1024 px, 4° of visual angle is about 107 px. We rounded this figure down and classified saccades less than 100 px long as short. A saccade was classified as long if it was over 600 px long; this was half the width of the space covered by the text.

Considering a fixation n, the algorithm treats it as a reading fixation in three different cases:

- fixation *n* is preceded by a short, horizontal forward saccade, i.e., oriented to the right in French, which is a left-to-right language (Equation 5). This is a normal reading saccade.
- fixation *n* is preceded by a long, almost horizontal backward saccade, corresponding to the so-called return sweep saccade going from the end of a line to the beginning of the next one (Equation 6)
- fixation *n* is preceded by a short, horizontal backward saccade preceded by a reading fixation *n*–1; this type of regressive saccade is quite common in reading (Equation 7).

All other fixations were classified as *scanning* (Equation 7). A fixation *n* resulting from a short, horizontal backward saccade preceded by a *scanning fixation n-1* is classified as *scanning* (Equation 8).

(5) $$\begin{array}{l} f\left(O(n),\;H(n),\;S(n)\right)=“Reading\prime \prime \;if\;O(n)>0\;and\;H(n)\\ \text{ < 45 }and\;S\text{(}n\text{) < 100} \end{array}$$

(6) $$\begin{array}{l} f\left(O(n),\;H(n),\;S(n)\right)=“Reading\prime \prime \;if\;O(n)<0\;and\;H(n)\\ \text{ < 45 }and\;S\text{(}n\text{) > 50\% }of\;text\;width\text{)} \end{array}$$

(7) f(O(n), H(n), S(n))=“Scanning″ otherwise

(8) $$\begin{array}{l} f\left(O(n),H(n),S(n)\right)=f\left(O(n-1),H(n-1),S(n-1)\right)\;if\;O(n)\\ \text{ < 0 }and\;H\text{(}n\text{) < 45 }and\;S\text{(}n\text{) < 100} \end{array}$$

This method does not depend on the content of the page, but only on the shape of the scanpath, which makes a difference with noisy data. The method used to record eye movements of subjects reading multi-line texts produces rather noisy data which does not allow the position of the eye to be determined with precision. Working from the shape of the scanpath instead of the content fixated is therefore necessary. Holmqvist et al. (2003) applied a similar method to identify scanning and reading fixations recorded when readers covered newspapers and net papers. They analyzed fixation data above 100 ms through a custom-made reading filter. Reading fixations were filtered if they were (1) before, between or after two successive forward saccades and (2) before and after return sweeps. Correction and backward saccades were not recognized. The fixations that were not filtered were labeled as scanning. Contrary to Holmqvist et al. (2003) filter, our classifier algorithm classify fixations that occurs after backward saccades.

### Design

The experiment used a full within-subjects design with 2 tasks (word-search, reading-for-comprehension), 2 banner animations (dynamic, static), and 3 positions (near, 0 px; intermediate, 40 px; far, 80 px) as experimental variables. These variables were counterbalanced in a Latin square design to produce 12 lists of stimuli and avoid any biases. Two trials per condition were assigned to the participants. In each list, 12 Web pages were assigned to the word-search task and 12 other Web pages to the reading-for-comprehension task. Web pages contained either a dynamic or a static advertisement positioned at 0, 40, or 80 px from the text. The 24 participants were randomly assigned to the 12 lists composed of 28 Web pages (4 training Web pages, 24 experimental Web pages).

### Experimental procedure

The experiment was run individually in an isolated and quiet workspace. First of all the participants read the instructions on the screen which described the reading tasks they were to perform: searching for a particular word in a text (*word-search task*) and reading the text carefully in order to provide a brief summary of the topic afterwards (*reading-for-comprehension task*). Then the experimenter asked the participants if they understood the instructions. The instructions for both tasks were developed in line with Carver's (1990) methodological recommendations. After calibration, the subjects performed the 4 training trials (2 trials for each task) and at the end the experimenter checked again that participants had understood the instructions. When everything had been checked, participants performed the 24 test trials. The two tasks were presented randomly across the trials.

On each trial of the word-search task a target word was displayed on the screen. Participants were instructed to memorize the word, then press the space bar and fixate a cross appearing for 1 s in the top center of the screen. The Web page was then displayed and the participant had to find the target word as quickly as possible. Once the target had been located participants had to fixate it for 1000 ms to trigger the end of that trial and start the next one. On each trial of the reading-for-comprehension task an instruction to read the text carefully was presented on the screen. Participants were then asked to press the space bar and to fixate a cross appearing for 1 s in the top center of the screen. This caused the Web page to be displayed and the participants could read the text. To complete the task participants had to close the browser and provide a brief written summary of the topic of the text in a dedicated area. The next trial started after their answer had been recorded.

The experimenter stayed with the participants throughout the training and experimental sessions to monitor the eye-tracking system. At the end, the participants answered a post-test questionnaire to assess their perception of the banners after which the experimenter explained the aim of the study to the participants and answered any questions.

## Data analyses

Analyses of variance for repeated measures (rm ANOVA) were conducted on 5 dependent variables: fixation duration, number of fixations, first-fixation duration, gaze duration and saccade amplitude, with a fixed significance threshold of *p* < 0.05. First-fixation duration was defined as the mean duration of the first 5 fixations. The objective was to investigate where subjects fixated when the webpage was first displayed and how the durations of these early fixations differed from those of the rest of the fixations. Gaze duration was the sum of fixation durations for an AOI (the text or the banner). The objective was to examine the total processing time for all the elements of the webpage. All analyses were corrected using Bonferroni *post-hoc* tests. A low cut-off of 100 ms and high cut-off of 500 ms were used for filtering fixations, these cut-offs corresponded to 2 SD above and below the average (i.e., 3.7% of outliers fixations were excluded). Outlanding saccades, i.e., saccades that landed outside the screen, were excluded from the analyses (0.24% of all saccades). After filtering the eye movement data the results of a Kolmogorov-Smirnov and Lilliefors test for normality were not significant (*KSL* ds > 0.05; *p* > 0.20) indicating that the data were normally distributed.

We defined 2 AOIs, one on the central text and one on the banner. The size of the AOI on the text was 430^*^565 px. The size of the advertisement AOI was the area of the banner (120^*^600 px).

## Results

**Figure 2:**
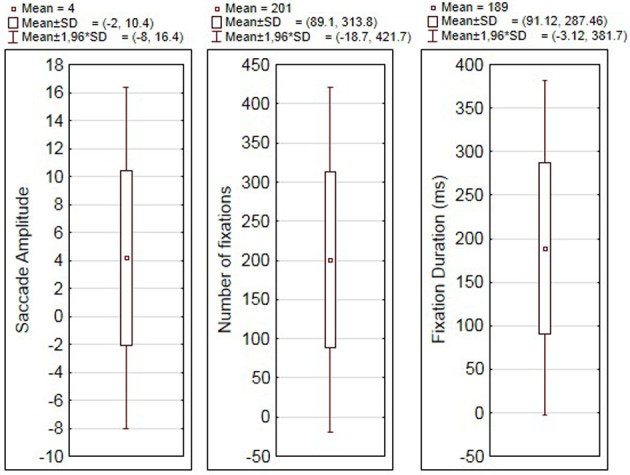
**Averages and standard deviations for fixation duration (ms), number of fixations and saccade amplitude (degrees of visual angle)**.

Table 1 summarizes the means and standard deviations for fixation durations, number of fixations and saccade amplitudes for all the experimental conditions (see also Figure 2).

**Table 1:** **Average fixation durations (ms), average number of fixations and average saccade amplitude (degrees of visual angle) by task, animation, and banner location for all participants**.

		**Animation**	**Distance of the banner from the target text**			
			**Near (0 px)**	**Intermediate (40 px)**	**Far (80 px)**	**Means**
Fixation duration (ms)	Reading-for-comprehension task	Dynamic	180 (38)	187 (35)	184 (41)	184
		Static	198 (38)	188 (39)	190 (33)	192
	Word-search task	Dynamic	193 (42)	193 (29)	190 (34)	192
		Static	179 (33)	185 (31)	188 (31)	184
	Means		188 ms	188 ms	188 ms	
Number of fixations	Reading-for-comprehension task	Dynamic	172 (96)	152 (86)	190 (92)	171
		Static	221 (91)	218 (80)	231 (93)	223
	Word-search task	Dynamic	244 (86)	251 (96)	215 (101)	237
		Static	163 (96)	152 (97)	161 (95)	159
	Means		200	193	199	
Saccade amplitude (° of visual angle)	Reading-for-comprehension task	Dynamic	4.2° (1.8)	4.5° (1.5)	3.9° (1.5)	4.2°
		Static	3.9° (1.4)	3.7° (0.9)	3.8° (1.2)	3.8°
	Word-search task	Dynamic	3.9° (0.8)	3.9° (1.0)	4.0° (1.3)	3.9°
		Static	3.1° (1.4)	4.4° (1.7)	4.3° (1.8)	3.9°
	Means		3.8°	4.1°	4.0°	

*Standard deviations are given in brackets*.

### Behavioral data: eye movements

#### Variation of eye data during reading activities

**Figure 3:**
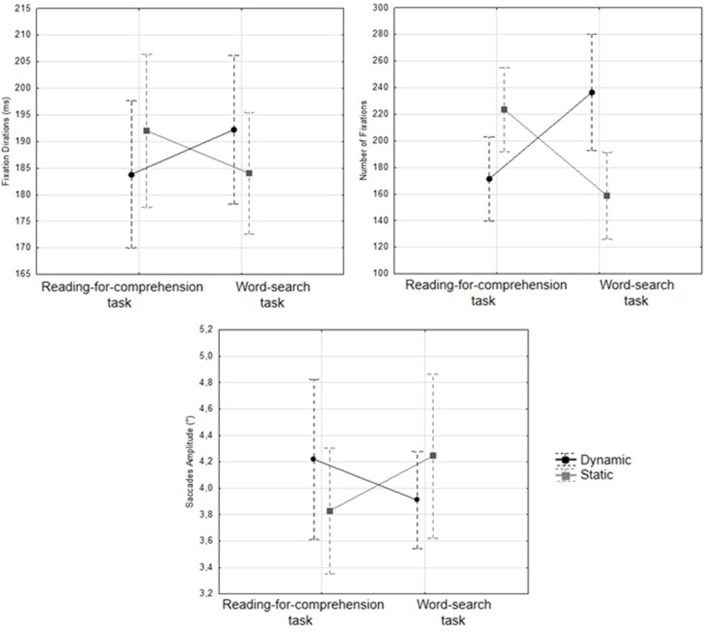
**Average fixation duration (ms), number of fixations and saccade amplitude (degrees of visual angle) according to task and animation**.

The following results consider all the eye movement data together regardless of where they terminated on the Web page (main text vs. banner). We hypothesized that overall eye movement data would be affected by banner animation. There were no significant main effects of the variables *Task*, *Animation*, and *Distance* on the eye movement metrics, all *F*s < 1. However, there was a significant interaction between *Task* and *Animation* for fixation duration [*F*_(1, 23)_ = 9.36, *p* < 0.010; η^2^ = 0.29, α = 0.05], number of fixations [*F*_(1, 23)_ = 41.76, *p* < 0.001; η^2^ = 0.64, α = 0.05], and saccade amplitude [*F*_(1, 23)_ = 7.76, *p* < 0.025; η^2^ = 0.25, α = 0.05]. During the word-search task, participants made more fixations (see Figure 3) [*F*_(1, 23)_ = 37.98, *p* = 0.008] and fixations were longer [*F*_(1, 23)_ = 6.48, *p* = 0.024] if the banner was dynamic. Although the effect did not reach significance, dynamic banners also tended to generate shorter saccades [*F*_(1, 23)_ = 2.70, *p* = 0.114]. The opposite pattern of results was found for the reading-for-comprehension task. When the banners were static Web pages received more fixations [*F*_(1, 23)_ = 11.71, *p* = 0.001] of longer duration, [*F*_(1, 23)_ = 4.94, *p* = 0.027] and saccades were shorter, [*F*_(1, 23)_ = 9.11, *p* = 0.020].

We did not obtain any significant results for either first-fixation durations or gaze durations, *p* = *ns*.

An ANOVA for *Trial Durations* (i.e., mean time required to complete the task) was also carried out to estimate readers' efficiency. Again the analyses revealed only one significant result: an interaction between *Task* and *Animation*, *F*_(1, 23)_ = 21.99, *p* < 0.001; η^2^ = 0.49, α = 0.05. During the word-search task, participants took longer to complete the task when the Web pages contained a dynamic banner, *F*_(1, 23)_ = 9.17, *p* = 0.017. The opposite result was found for the reading-for-comprehension task: completion times were longer when the banner was static, *F*_(1, 23)_ = 12.00, *p* = 0.019.

#### Advertisement and areas of interest (AOIs)

**Figure 4:**
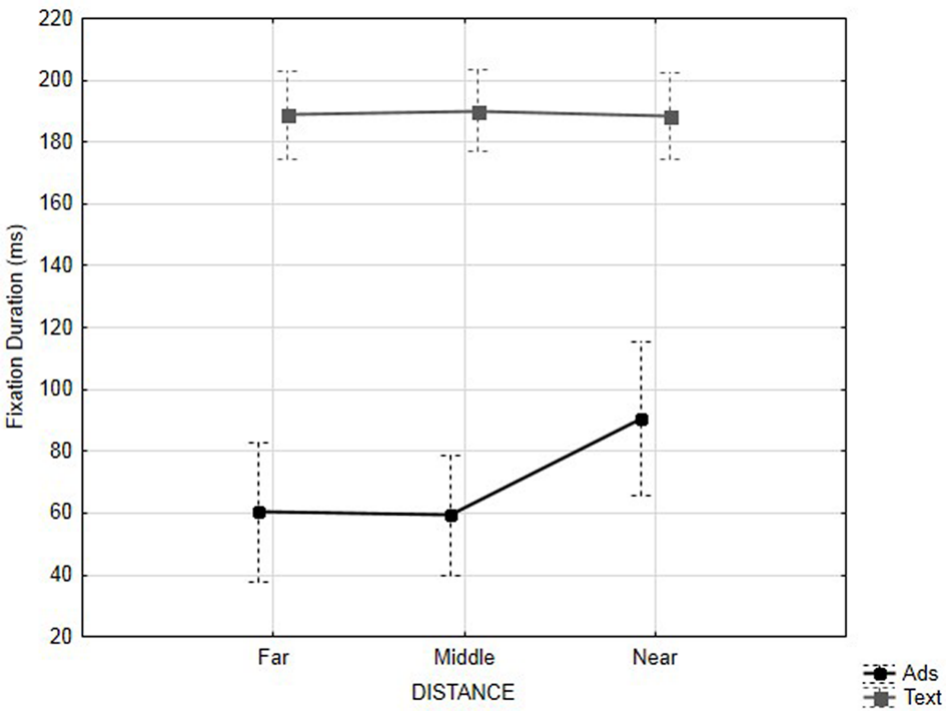
**Average fixation duration (ms) according to distance and zone (AOIs)**.

In order to investigate the banner blindness effect, an ANOVA was carried out for all eye movement data from two AOIs: one defined on the banner and another one on the central text. We defined another factor *Zone* to investigate differences between the two AOIs. We weighted the fixation durations and the number of fixations according to the size of the AOIs. The results showed that the central text received significantly longer fixations than the banner, *F*_(1, 23)_ = 176.61, *p* < 0.001; η^2^ = 0.88, α = 0.05. It also attracted significantly more fixations, *F*_(1, 23)_ = 324.89, *p* < 0.001; η^2^ = 0.93, α = 0.05. The position of the banner also affected fixation duration, *F*_(2, 46)_ = 3.39, *p* < 0.050; η^2^ = 0.13, α = 0.05. Banners near the central text received longer fixations than banners at an intermediate distance or far from the text, *F*_(1, 23)_ = 5.14, *p* = 0.000. There was no significant difference between intermediate and far banners, *p* = ns. There was a significant interaction between *Zone* and *Distance* on fixation durations, *F*_(2, 46)_ = 4.34, *p* < 0.025; η^2^ = 0.16, α = 0.05. Fixation durations for the central text did not vary according to the distance of the banner from the text, all *F*s < 1. However, banners near the central text received longer fixations than banners at an intermediate distance or far from the text, *F*_(1, 23)_ = 4.78, *p* = 0.020 (see Figure 4). The results also indicated a three-way interaction with *Zone, Task and Animation*, *F*_(1, 23)_ = 40, 20, p < 0.001; η^2^ = 0.64, α = 0.05. However, the number of fixations only varied on the central text, during the word-search task with the text received more fixations when the advertisement was dynamic, *F*_(1, 23)_ = 11.08, *p* = 0.000, but during the reading-for-comprehension task the text received more fixations when the banner was static, *F*_(1, 23)_ = 35.40, *p* = 0.000.

#### Post-test questionnaire and visual strategies

**Figure 5:**
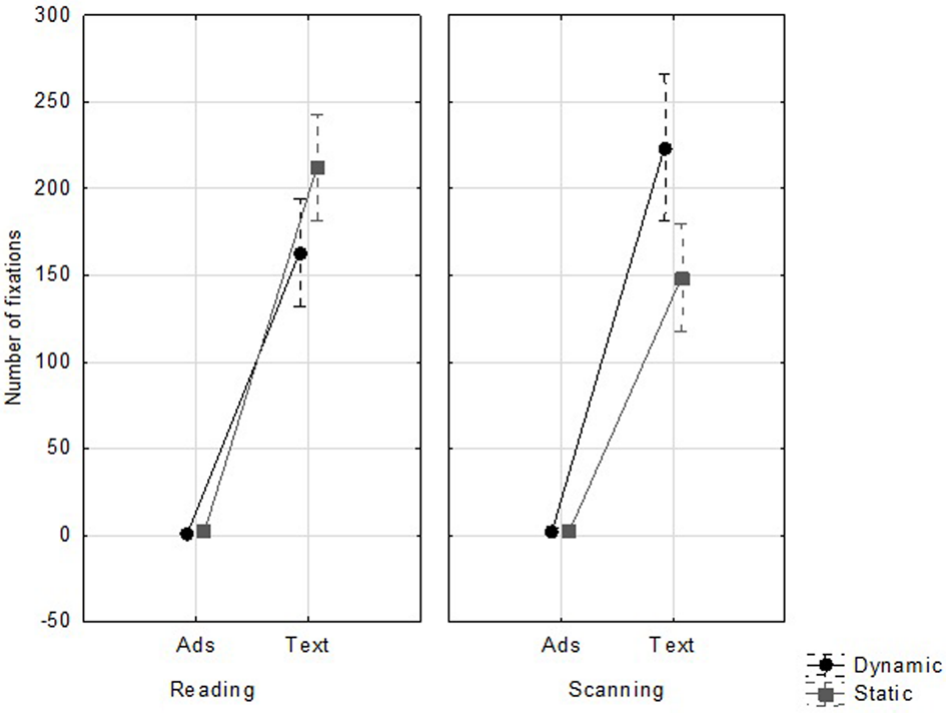
**Average number of fixations (ms) according to task, animation and zone (AOIs)**.

The post-test questionnaire was used to investigate participants' subjective perception of shifts of attention toward the banners and how they thought they had been affected by the banners. The higher the score, the more attention grabbed and the more distraction felt. Of the 24 participants, 13 (54%) reported that they did not pay attention to the banner and were not affected by the banners (*M* = 1.4; *SD* = 0.46) (see Figure 5).

ANOVAs were carried out for the eye movement data from the 13 participants who reported that they had not paid any attention to the banners, to investigate possible automatic and unconscious shifts of attention (see Table 2). These participants covered the Web pages with more fixations when they contained a dynamic banner, *F*_(1, 12)_ = 7.32, *p* < 0.025; η^2^ = 0.38, α = 0.05. A significant interaction between *Task* and *Animation* [*F*_(1, 12)_ = 14.30, *p* < 0.010; η^2^ = 0.54, α = 0.05] indicated that dynamic banners only affected the number of fixations during the word-search task, *F*_(1, 12)_ = 20.05, *p* = 0.005. There was also an interaction between the *Animation* and *Distance* of the banners for fixation durations during the reading-for-comprehension task, *F*_(1, 12)_ = 3.56, *p* < 0.050; η^2^ = 0.23, α = 0.05. The far (80 px) banners generated longer fixations when they were static, *F*_(1, 12)_ = 5.40, *p* = 0.036.

**Table 2:** **Average fixation durations (ms), average number of fixations and average saccade amplitude (degrees of visual angle) by task, the animation and banner location for the 13 participants who reported that they were not affected by the advertisements**.

		**Animation**	**Distance of the banner**			
			**Near (0 px)**	**Intermediate (40 px)**	**Far (80 px)**	**Means**
Fixation duration (ms)	Reading-for-comprehension task	Dynamic	207 (36)	196 (30)	187 (38)	197
		Static	200 (32)	196 (41)	212 (32)	203
	Word-search task	Dynamic	199 (30)	202 (25)	199 (43)	200
		Static	196 (36)	193 (30)	190 (27)	193
	Means		201	197	197 ms	
Number of fixations	Reading-for-comprehension task	Dynamic	229 (93)	179 (88)	179 (78)	196
		Static	232 (80)	204 (70)	203 (66)	213
	Word-search task	Dynamic	241 (98)	243 (93)	238 (79)	241
		Static	169 (98)	143 (82)	165 (97)	159
	Means		218	192	196	
Saccade amplitude (° of visual angle)	Reading-for-comprehension task	Dynamic	3.8° (1.2)	4.1° (1.2)	3.9° (1.5)	3.9°
		Static	3.6° (0.8)	3.6° (0.7)	3.6° (0.8)	3.6°
	Word-search task	Dynamic	3.9° (0.8)	3.8° (0.8)	4.1° (1.3)	3.9°
		Static	3.7° (0.8)	4.4° (1.5)	3.9° (1.2)	4.0°
	Means		3.8°	4.0°	3.9°	

*Standard deviations are given in brackets*.

We did not obtain any significant results for the saccade amplitudes of the 13 participants, *p* = *ns*.

### Classifier algorithm: proportions of *scanning* and *reading*

An algorithm was developed to explore how visual strategies vary according to the text layout and how advertising banners affect visual strategies according to the processing depth and explore how ads generate task-switching. This algorithm classified all fixations as *Scanning* or *Reading* according to the saccade that preceded the fixation. Logically, more scanning fixations should be found in the search task and more reading fixations in the reading-for-comprehension task. This classifier algorithm has been applied on fixations data (i.e., after an event detection has been applied on eye samples for detecting fixation and saccade). A lots of event-detection algorithms have been used in the eye-tracking literature, but an interesting one which might improve also the accuracy of our algorithm has been recently developed by Nyström and Holmqvist (2010) for fixation, saccade and glissade detection. It seems fairly robust and has addressed problems which affected other event-detection algorithms and would be of interest in this context.

We computed a scanning rate for each condition and each participant (Table 3). The results suggested that participants switched between the strategies during both tasks. About half the fixations during the reading-for-comprehension task were classified as *Scanning* (*M* = 50.67, *SD* = 8.98). The proportion of fixations classified as scanning was slightly larger for the word-search task (*M* = 52.25, *SD* = 8.45). There was considerable variability in strategy between the participants across conditions (*M* = 51.5, *SD* = 11.34). For example, Participant 17 used a scanning strategy much more than Participants 4 and 15 (64 vs. 36% averaged across conditions).

**Table 3:** **Proportion of *Scanning* fixations (as %) for each participant averaged over conditions according to the classifier algorithm**.

**Part.**	**Word-search task**						**Means**	**Reading-for-comprehension task**						**Means**	**Means**
	**Dynamic**			**Static**			**scan.**	**Dynamic**			**Static**			**read.**	
	**Far**	**Intermediate**	**Near**	**Far**	**Intermediate**	**Near**		**Far**	**Intermediate**	**Near**	**Far**	**Intermediate**	**Near**		
1	48	44	47	46	38	45	44	38	42	37	39	34	33	37	41
2	57	46	61	67	51	59	57	51	42	42	37	41	58	45	51
3	74	45	53	67	67	66	62	46	48	56	47	67	51	52	57
4	37	31	33	45	31	43	37	30	51	29	36	35	30	35	36
5	42	42	40	47	59	55	48	42	34	44	47	53	55	46	47
6	56	52	52	63	73	63	60	49	46	51	57	64	65	55	58
7	32	36	38	54	33	64	43	32	36	40	47	31	44	38	41
8	47	50	47	65	63	60	55	49	54	50	52	58	63	54	55
9	42	45	42	57	54	58	50	52	40	47	40	62	57	50	50
10	44	41	50	44	41	58	46	39	44	38	43	38	39	40	43
11	43	45	34	37	41	43	40	38	57	40	34	53	35	43	41
12	52	59	56	68	55	54	57	47	56	58	49	73	54	56	57
13	56	62	67	68	66	55	62	44	47	51	52	43	41	46	55
14	79	78	67	64	69	56	69	53	42	67	64	60	66	59	64
15	34	39	34	30	38	39	36	41	27	35	38	38	40	37	36
16	64	49	55	63	70	59	60	55	57	48	86	55	52	59	60
17	63	55	62	71	53	60	61	57	72	59	51	71	92	67	64
18	50	48	50	52	48	46	49	44	51	55	51	48	68	53	51
19	48	41	45	71	53	75	55	44	37	37	66	65	68	53	54
20	49	51	44	65	57	49	52	49	62	56	64	63	59	59	55
21	53	54	66	63	57	60	59	62	62	61	73	67	66	65	62
22	42	46	42	56	46	52	47	40	55	54	52	54	62	53	50
23	42	49	60	45	44	50	48	55	50	55	54	40	52	51	50
24	57	56	61	50	58	61	57	65	57	56	65	72	62	63	60
Means	50	49	50	57	53	55		47	49	49	52	54	55		

We carried out a Friedman ANOVA on the proportion of fixations assigned to each strategy. Proportions of scanning were compared by *Task* and *Animation*. Significant differences were found in use of the scanning strategy across the conditions, χ^2^_(11, 23)_ = 30.00, *p* < 0.010. For both the word-search and reading-for-comprehension tasks, Tukey's HSD *post-hoc* test revealed that participants used a *Scanning* strategy significantly more when the banner was static (*p* < 0.050). When the advertising banner was static participants switched to a *Scanning* strategy more often during the reading-for-comprehension task than during the word-search task (*p* < 0.010).

## Discussion

The impact of online advertisement has been the topic of research for many years. The theoretical debate has contrasted top–down and bottom–up processing (Theeuwes, 1994; Theeuwes and Burger, 1998; Drèze and Hussherr, 2003; Stenfors et al., 2003; Simola et al., 2011) and overt and covert shifts of attention (Benway and Lane, 1998; Itti and Koch, 2000). In the present work we investigated how the animation and placement characteristics of advertising banners affected readers' eye movements and thus their cognitive states, during two different reading activities. Previous studies of visual processing activities using statistical models suggested that eye movements reflect readers' cognitive states (Carver, 1990; Rayner and Pollatsek, 1992; Rayner, 1995, 1998; Simola et al., 2008; Cole et al., 2011; Lemaire et al., 2011; Henderson et al., 2013). We predicted that the closer the advertisement, the more difficult participants would have with task processing. We also hypothesized that animated banners would be more distracting than static advertisements. We predicted that the banners would have a stronger effect during the word-search task, but that participants would experience “banner blindness” during the reading-for-comprehension task. We recorded the eye movements of participants performing both word-search and reading-for-comprehension tasks and investigated transitions between visual strategies with the help of a classifier algorithm that differentiates *scanning* fixations from *reading* fixations.

The results revealed that readers' eye movements were affected differently by the characteristics of the advertising banners during the word-search and reading-for-comprehension tasks. When participants were performing the word-search task, the eye movement data showed smaller fixation durations, fewer fixations, shorter saccades and less efficiency when the banners were dynamic rather than static. During the reading-for-comprehension task performance was worse when the banners were static. On both the word-search and reading-for-comprehension tasks, the variations in the number of fixations only applied to the central text. The results also indicated that the central text received longer fixations than the banner and that variations in fixation durations for the banner only occurred when it was near the central text. Although 54% of the participants reported that they had not paid attention to the banners the results showed they were affected by dynamic banners during the word-search task and by the distant (80 px) static banners when performing the reading-for-comprehension task. The results of the strategy classification algorithm suggested that when readers were performing the word-search task they switched from a scanning strategy to a reading strategy more often if the banner was dynamic, whereas when they were performing the reading-for-comprehension task, they switched from a reading strategy to a scanning strategy more often if the banner was static.

These results have implications for understanding how online advertising banners grab users' attention. They strongly suggest that advertisements affected users in a bottom–up manner. The banners, as salient elements of the Web pages, automatically generated shifts of attention toward them. Although in the current study most of the attentional shifts were covert, these data also provide evidence supporting overt attention theories (Simola et al., 2011). Shifts of attention toward the advertisements were sometimes accompanied by an eye movement. Our comparative analysis of the use of scanning and reading strategies is consistent with previous work suggesting that advertisements have a negative impact on users' performance (Diaper and Waelend, 2000; Burke et al., 2005; Zhang, 2006). Whilst performing the word-search task, participants appear to have slowed down their reading rate more often when the banner was dynamic. During the reading-for-comprehension task, readers seemed to experience more difficulty maintaining a consistent reading rate and switched to a scanning strategy more often when the banner was static. Nevertheless, we expected both static and dynamic banners to affect users more during the word-search task than during the reading-for-comprehension task. Our results showing that dynamic banners had a greater impact on the word-search task than static banners are consistent with previous issues (Simola et al., 2011). However, nothing in the literature explains the interaction between task-type and animation. One possible explanation is that the reading-for-comprehension task was highly demanding, leaving fewer attentional resources available for organizing the sharing of attentional capacity between task processing and banner processing. Participants may also have used strategies actively to ignore the banners. The higher salience of the animated advertisements may have made them easier to ignore. Contrary to previous research which suggested that online advertisements have more impact during tasks requiring low-level information processing, such as the word-search task (Burke et al., 2005; Pagendarm and Schaumburg, 2006; Simola et al., 2011), we found that advertisements affected performance on both tasks. Participants were more affected by dynamic advertisements whilst performing the word-search task, but more disrupted by static advertisements whilst reading for comprehension.

Our data also suggest that readers were not completely able to ignore the advertisements, although banners were generally not fixated directly in both tasks. The number of fixations on the text varied with task and animation. Shifts of attention toward the banner were mostly covert. However, fixation durations on advertisements may imply that sometimes participants glanced briefly at the banners. It is possible that participants used banner blindness strategies when the banners were distant from the central text, but without complete success. The data from participants who claimed that they were not affected by the banners are consistent with findings from Theeuwes and Burger (1998). These authors suggested that banner blindness only occurs when users are aware of the distractors and their features, and when distractors do not vary randomly during the task. In this study advertisements varied unpredictably and participants were not warned about them, which may explain why all the participants were disturbed by them. All our participants were experts Internet users so our findings provide support for Zhang's (2006) assertion that users cannot habituate to online advertisements.

From a practical standpoint, the current work has implications for the design of Web interfaces and could guide Web developers and advertisers in their choice of advertising banners. Banners which are well separated from target material would be preferred by Web developers seeking to limit the impact of advertisements on users and to offer more user-friendly Web interfaces. Whenever possible (depending of the device size) advertisers might prefer to display ads closer to the main content of the Web pages as closer banners attracted longer fixation durations. However, it should be noted that the number of fixations did not vary with distance from the target text. The decision about use of animation might depend on the aim of the Web developer or advertiser; it could also depend on the task for which the Web interface was designed. For tasks which require only low-level cognitive processing, static advertisements might be preferred by Web designers although advertisers would choose dynamic banners. The opposite pattern of preferences would probably apply to tasks requiring greater depth of processing. The present study has demonstrated that eye movements and visual strategies are affected by online advertisements underlining that users' cognitive states are also affected by advertisements. The choice of the type of online advertisement depends on the objective. In future work, it would be interesting to replace the right-hand side banners with another type of advertisement such as pop-ups.

### Conflict of interest statement

The authors declare that the research was conducted in the absence of any commercial or financial relationships that could be construed as a potential conflict of interest.

## Footnotes

^1^Frequency per million of the lemma in the corpus of books: it corresponds to the sum of the frequencies of the inflected forms of each lemma in the corpus Frantext, normalized by dividing by 14.8 (the original corpus contains 14.7 million occurrences).
